# DNA methylation in cord blood in association with prenatal depressive symptoms

**DOI:** 10.1186/s13148-021-01054-0

**Published:** 2021-04-12

**Authors:** Theodora Kunovac Kallak, Emma Bränn, Emma Fransson, Åsa Johansson, Susanne Lager, Erika Comasco, Robert Lyle, Alkistis Skalkidou

**Affiliations:** 1 Department of Women’s and Children’s Health, Uppsala University, Uppsala, Sweden; 2 Department of Immunology, Genetics and Pathology, Science for Life Laboratory, Uppsala University, Uppsala, Sweden; 3 Department of Neuroscience, Science for Life Laboratory, Uppsala University, Uppsala, Sweden; 4 Department of Medical Genetics and Norwegian Sequencing Centre (NSC), Oslo University Hospital, Centre for Fertility and Health, Norwegian Institute of Public Health, Oslo, Norway

**Keywords:** CBCL, Cord blood, DNA methylation, EPDS, EWAS, Prenatal depressive symptoms, Women

## Abstract

**Background:**

Prenatal symptoms of depression (PND) and anxiety affect up to every third pregnancy. Children of mothers with mental health problems are at higher risk of developmental problems, possibly through epigenetic mechanisms together with other factors such as genetic and environmental. We investigated DNA methylation in cord blood in relation to PND, taking into consideration a history of depression, co-morbidity with anxiety and selective serotonin reuptake inhibitors (SSRI) use, and stratified by sex of the child. Mothers (*N* = 373) prospectively filled out web-based questionnaires regarding mood symptoms and SSRI use throughout pregnancy. Cord blood was collected at birth and DNA methylation was measured using Illumina MethylationEPIC array at 850 000 CpG sites throughout the genome. Differentially methylated regions were identified using Kruskal–Wallis test, and Benjamini-Hochberg adjusted *p*-values < 0.05 were considered significant.

**Results:**

No differential DNA methylation was associated with PND alone; however, differential DNA methylation was observed in children exposed to comorbid PND with anxiety symptoms compared with healthy controls in *ABCF1* (log twofold change − 0.2), but not after stratification by sex of the child. DNA methylation in children exposed to PND without SSRI treatment and healthy controls both differed in comparison with SSRI exposed children at several sites and regions, among which hypomethylation was observed in CpGs in the promoter region of *CRBN (*log2 fold change − 0.57), involved in brain development, and hypermethylation in *MDFIC* (log2 fold change 0.45), associated with the glucocorticoid stress response.

**Conclusion:**

Although it is not possible to assess if these methylation differences are due to SSRI treatment itself or to more severe depression, our findings add on to existing knowledge that there might be different biological consequences for the child depending on whether maternal PND was treated with SSRIs or not.

**Supplementary Information:**

The online version contains supplementary material available at 10.1186/s13148-021-01054-0.

## Introduction

Common complications during pregnancy regard poor mental health. Maternal prenatal symptoms of depression (PND) prevalence range from 4 to 29%, depending on context and assessment methods [1], with the potential to affect the development of the child [2]. In a previous study from our group, PND (compared with postpartum depression) was particularly influential on child behavioral problems [3]. Furthermore, children exposed to maternal depression during pregnancy are reported to be at increased risk of altered brain development [4] as well as cognitive difficulties [5, 6]. A sex specific impact from exposure to PND has also been suggested, such as larger effect on cognitive development in boys and on emotional development in girls [5].

Anxiety is a common mental health problem in pregnant women. High rates of anxiety symptoms have been reported among pregnant women, varying between 4.4 and 39% [7]. Anxiety is often comorbid with depression and depression in the peripartum period is sometimes viewed as comprising much of the anxiety phenotype [5]. Depressive symptoms in women with a history of depression [8, 9] or in women with co-morbid antenatal symptoms of anxiety [10] are thus of special interest when considering eventual differences in pathogenesis and consequences. Finally, treatment with selective serotonin reuptake inhibitor (SSRI) has been shown to be associated with biological correlates such as altered cortico-releasing hormone levels and differential gene expression in the placenta [11, 12].

The link between maternal mental health problems and child development is complex and includes genetic factors, some with possibly pleiotropic effects, as well as environmental and family factors [13], which could impact on fetal development through many biological pathways. Greater levels of stress hormones and inflammation have been reported in cord blood of preterm neonates exposed to poor maternal mental health [14]. Apart from direct impact on fetal biological systems, alterations resulting from maternal depression during pregnancy are thought to induce epigenetic changes in developmental pathways. A hypothesis of epigenetic programming due to maternal behavior has evolved and includes research on experimental animal models [15]. There is also a growing body of human studies investigating the impact of prenatal exposure to maternal depressed mood in infants [16–18].

The focus of these epigenetic studies has been on DNA methylation, which is a chemical signature that can be alongside DNA replication, through transfer of methylation groups by DNA methyltransferase, and influence gene expression, thus representing a candidate mechanism for long-term effects of environmental exposure on phenotype [19]. The role of epigenetics in neural development has been mainly explored in epigenome-wide association studies (EWAS). In fetal brain samples, associations have been demonstrated between DNA methylation and brain development, these specific sites have also been seen to be schizophrenia risk loci [20]. The use of the more accessible surrogate tissue such as blood for these investigations has also been evaluated [21, 22]. Common environmental exposures could cause both epigenetic modifications in umbilical cord blood and brain, affecting the child neural development, for example, paracetamol usage during pregnancy is associated with attention deficit/hyperactivity disorder (ADHD) (reviewed by Bauer et al. [23]) and long-term exposure to paracetamol has shown altered DNA methylation in cord blood in children who later develop ADHD [24].

Studies on how fetal exposure to depressive symptoms alone, or with comorbidities, with or without SSRI treatment, affects DNA methylation have shown varied results, largely due to different methodologies and cohorts [16, 25–27]. Of special interest is the difference in cell composition between cord blood and whole blood from adults [28, 29] and previous studies on the association between PND and DNA methylation in cord blood have, however, not taken this into account [16, 17]. The sex of the child may also moderate the epigenetic influence by PND, since there are differences between sexes in DNA methylation patterns [30].

Altered DNA methylation in cord blood has been observed in children exposed to anxiety and also exposure to SSRIs has been associated with hypomethylation in *ZNF575*, a gene whose functions are largely unknown [16]. However, this study did not consider comorbidity of PND with anxiety nor did it compare exposure to SSRI with exposure to untreated PND [16]. A review by Viuff and colleagues assessed studies investigating the effect of SSRIs on cord blood DNA methylation and showed the need of new investigations using wide-range DNA methylation analysis, validation of exposure to SSRI, and analyses combining PND with other mental illness [31].

The aim of the present study was therefore to investigate the possible association between PND and DNA methylation in cord blood. Secondary aims, unique to this investigation, were to reassess the association among different subtypes of PND and to investigate the effect of sex. Specifically, subtypes were those with a history of depression or co-morbidity with symptoms of anxiety in comparison with PND alone, and also those treated with SSRIs in comparison with untreated PND and healthy controls.

## Results

### Demographics

Characteristics of the participating mothers are presented in Table 1. A university level education was less frequent among mothers with PND. Comorbid anxiety was reported in over 57 percent of the women with PND. The children of mothers with and without PND had not significantly different birth weight. There were no significant difference in the proportion of mothers born in Scandinavia between any of the analyzed study groups. Children born by women with PND had slightly shorter gestational length.

**Table 1:** Characteristics of participating women by maternal pregnancy depressive symptoms, *N* = 373

	Healthy controls *N* = 195	Prenatal depressives symptoms *N* = 178	*p*
*Maternal characteristics*			
Age, median (range)	31.8 (23.0–48.0)	30.0 (19.0–45.0)	0.063
Born in Scandinavia, *n* (%)^a^	183 (93.8)	160 (89.9)	0.277
Educational attainment, *n* (%)^a^			
University level	169 (86.7)	110 (62.9)	0.000
Primipara, *n* (%)	103 (52.8)	83 (46.6)	0.138
Body mass index (BMI), median (range)^b^	22.6 (17.8–34.6)	22.9 (17.6–38.9)	0.342
Vaginal delivery, *n* (%)	154 (79.0)	135 (75.8)	0.370
History of depression, *n* (%)	0 (0)	132 (74.2)	0.000
Anxiety during pregnancy	0 (0)	101 (56.7)	0.000
EPDS^c^, scores at gestational week 17, median (range)	2 (0–11)	13 (0–23)	0.000
EPDS^c^, scores at gestational week 32, median (range)	2 (0–11)	13 (2–21)	0.000
*Child characteristics*			
Sex of the child			
Boy	97 (49.7)	92 (50.2)	0.678
Girl	98 (50.3)	84 (49.8)	
Gestational age (days), median (range)	281 (245–296)	280 (250–296)	0.044
Birth weight (kg) median (range)	3.63 (1.62–5.0)	3.65 (2.27–4.93)	0.722

^a^Data missing on 11 participants

^b^Data missing on 10 participants

^c^Edinburgh Postnatal Depression Scale

### Cord blood DNA methylation in relation to maternal depression symptoms during pregnancy

After adjusting for potential confounding factors and controlling for multiple testing, in all group analyses, the number of DM CpGs between all study groups are presented in Table 2 and the data are displayed as quantile–quantile (QQ)-plots in Additional file 1: Fig. 1. In our main analysis, no CpG sites in cord blood were associated with maternal PND in comparison with healthy controls (HC). Further, there were no DM CpGs when comparing PND with or without history of depression to each other or HC. On the other hand, PND comorbid with anxiety showed a total of two DM CpGs in comparison with HC: at cg05385119 located just upstream of the ATP Binding Cassette Subfamily F Member 1 gene (*ABCF1*) (log twofold change − 0.21) and at cg00017362 (log twofold change 0.18) located upstream of Homo sapiens integrator complex subunit 10 gene (*INTS10*) (Table 3, Additional file 2: Fig. 2). Stratification by sex of the child did not reveal any DM between study groups (Table 2).

**Table 2:** Overview of the number of differentially methylated CpG sites in different group comparisons

		Whole				Girls				Boys			
Group 1	Group 2	n1	n2	pa	pu	n1	n2	pa	pu	n1	n2	pa	pu
Healthy control	Prenatal depressive symptoms	194	165	0	49,385	98	77	0	43,762	96	88	0	35,603
History and prenatal depressive symptoms	Prenatal depressive symptoms only	147	32	0	37,873	65	18	0	43,919	82	14	38	39,150
History and prenatal depressive symptoms	Healthy control	147	194	0	44,372	65	98	0	37,458	82	96	0	35,522
Prenatal depressive symptoms only	Healthy control	32	194	0	45,043	18	98	0	47,277	14	96	6	39,941
Anxiety and prenatal depressive symptoms	Prenatal depressive symptoms only	95	70	0	56,366	41	36	0	46,103	54	34	0	44,805
Anxiety and prenatal depressive symptoms	Healthy control	95	194	**2**	44,230	41	98	0	39,519	54	96	0	36,549
Prenatal depressive symptoms only	Healthy control	70	194	0	62,013	36	98	0	50,096	34	96	0	41,260
Healthy control	Prenatal depressive symptoms without SSRI	194	149	0	48,338	98	71	0	41,955	96	78	0	34,973
Healthy control	Prenatal depressive symptoms with SSRI	194	13	**14**	44,881	98	4	**109**	48,451	96	9	**14**	49,785
Prenatal depressive symptoms without SSRI	Prenatal depressive symptoms with SSRI	149	13	**14**	41,708	71	4	**131**	40,940	78	9	**12**	50,239

pa = number of CpGs significant with *p*-value adjusted for multiple testing and for sample plate batch, sentrix position on the chip, granulocyte cell estimate, nucleated red blood cell estimate,  CD8T-cell estimate, maternal age at partus, pre-pregnancy body mass index, maternal place of birth, parity, gestational age, delivery mode, and maternal education

pu = number of differentially methylated CpGs with an unadjusted significant *p*-value

**Fig. 1:**
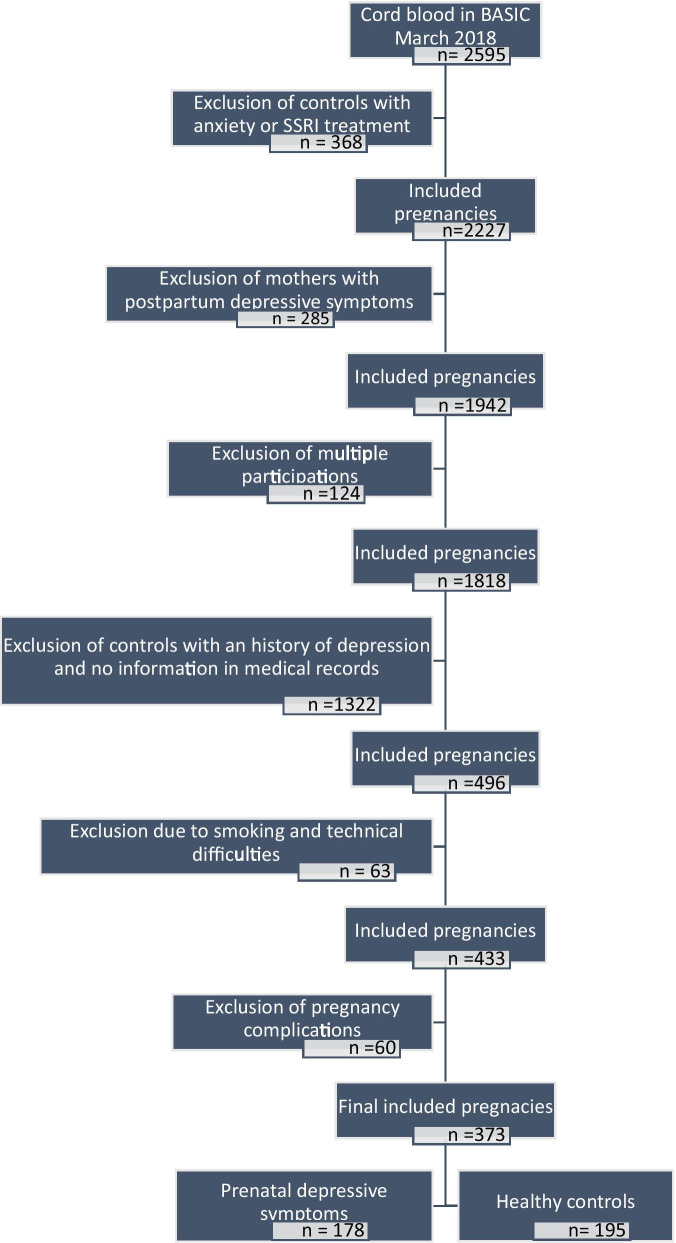
Flowchart describing selection of mothers for DNA methylation analysis from the BASIC study cohort

**Fig. 2:**
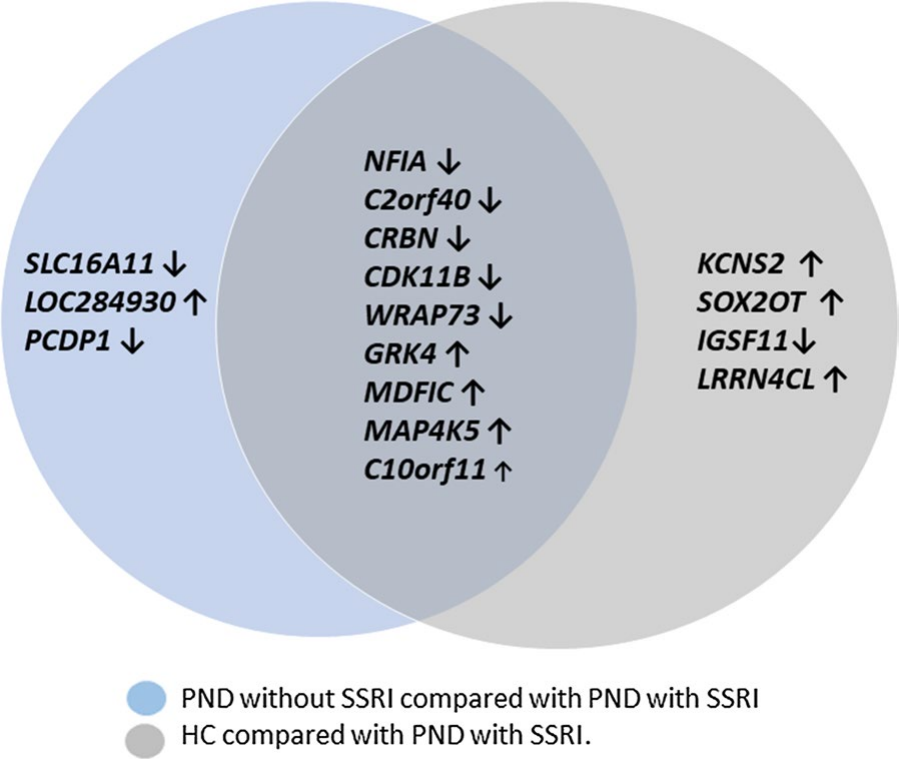
Venn diagram displaying genes with CpGs differently methylated in cord blood from children with mothers experiencing prenatal depressive symptoms (PND) without selective serotonin receptor inhibitor (SSRI) treatment compared with PND with SSRI treatment (blue) and healthy controls compared with PND with SSRI treatment (grey). Arrow pointing upwards indicates higher DNA methylation in the PND without SSRI group (blue) or HC (grey) in comparison with PND with SSRI treatment while arrows pointing downwards indicate lower DNA methylation

Additionally, PND with or without SSRI treatment was tested (Table 3, Additional file 2: Fig. 2). A total of 14 CpGs were DM among PND cases without SSRIs compared with those on SSRI treatment, while HC showed 14 CpG sites to be DM compared PND on SSRI treatment. Nine of the CpG sites identified coincided in both comparisons, meaning they were DM in both PND without SSRI and HC compared with PND on SSRIs, Fig. 2. Pathway enrichment analysis did not show any association between the DM genes and known pathways. Stratified analyses based on the sex of the child showed, among girls, 131 DM CpGs sites in PND without SSRIs compared with PND treated with SSRI, while HC showed 109 CpG sites to be DM compared with PND treated with SSRIs. In boys, PND without SSRIs compared with PND treated with SSRIs showed 12 CpGs to be DM, while 195 CpG sites were DM healthy controls in comparison with PND on SSRIs.

**Table 3:** Differentially methylated CpGs

Group 1	Group 2	Gene	Location	Probe	Log2 fold change	Mean SD	Adjusted *p*-value	Regulatory feature
Anxiety and prenatal depressive symptoms	Healthy control	ATP Binding Cassette Subfamily F Member 1(ABCF1)	chr6. 30539063	cg05385119	− 0.21	− 3.80 (0.35), − 3,60(0,26)	0.037	Promoter Associated
		Located upstream of Homo sapiens integrator complex subunit 10 gene (INTS10)	chr8. 19711803	cg00017362	0.18	3.04(0,29), 2,89(0.26)	0.037	
Healthy controls	Prenatal depressive symptoms with SSRI	Nuclear Factor I A (NFIA)	chr1. 61549982	cg18946602	− 0.91	2.05(0.50), 2.82 (0.96)	0.005	
		Chromosome 2 open reading frame 40 (C2orf40)	chr2 106682209	cg17161250	− 0.71	− 3.83(0.38), − 3.14(1.10)	0.004	Unclassified cell-type specific
		Immunoglobulin Superfamily Member 11 (IGSF11)	chr3 118864895	cg20675391	− 0.70	3.74(0.43), 4.35(1.11)	0.024	Unclassified
		Cereblon (CRBN)	chr3 3221595	cg20787969	− 0.57	− 4.39(0.26), − 3.83(1.39)	0.020	Promoter Associated
		Cyclin-Dependent Kinase 11B (CDK11B)	chr1 1655867	cg00669623	− 0.29	− 3.32 (0.12), − 3.04(0.74)	0.021	Promoter Associated
		WD Repeat Containing. Antisense To TP73 (WRAP73)	chr1 3551807	cg04386615	− 0.54	3.64(0.37), 4.15(0.46)	0.048	
		G Protein-Coupled Receptor Kinase 4 (GRK4)	chr4 3033304	cg03178823	0.43	2.47(0.20), 1.99(0.93)	0.010	
		MyoD Family Inhibitor Domain Containing (MDFIC)	chr7 114562316	cg02239805	0.44	− 1.52(0.09), − 1.96(1.42)	0.010	Promoter Associated
		Potassium Voltage-Gated Channel Modifier Subfamily S Member 2 (KCNS2)	chr8 99441950	cg02143496	0.49	3.19(0.29), 2.71(0.88)	0.010	
		LRRN4 C-Terminal Like (LRRN4CL)	chr11 62455369	cg03353995	0.49	− 2,38(0.32), − 2,80(0.33)	0.041	
		Mitogen-Activated Protein Kinase Kinase Kinase Kinase 5 (MAP4K5)	chr14 50999680	cg21062897	0.49	− 0.85 (0.15),—1.33(1.61)	0.018	Promoter Associated
			chr3 169476270	cg16056894	0.56	− 1.33(0.35), − 1.82(0.67)	0.029	
		SOX2 Overlapping Transcript (SOX2OT)	chr3 181444989	cg17202313	0.57	− 3.76(0.36), − 4.24(0.70)	0.030	Unclassified cell-type specific
		Chromosome 10 open reading frame 11 (C10orf11)	chr10 77871958	cg24280832	0.98	1.54(0.39), 0.51(1.72)	< 0.000	
		Nuclear Factor I A (NFIA)	chr1 61549982	cg18946602	− 0.80	2.11(0.56), 2.82(0.95)	0.041	
Prenatal depressive symptoms without SSRI	Prenatal depressive symptoms with SSRI	Chromosome 2 open reading frame 40 (C2orf40)	chr2 106682209	cg17161250	− 0.72	− 3.87(0.41), − 3.14(1.10)	0.003	Unclassified cell-type specific
		Cereblon (CRBN)	chr3 3221595	cg20787969	− 0.57	− 4.38(0.25), − 3.83(1.39)	0.026	Promoter Associated
		Primary Ciliary Dyskinesia Protein 1 (PCDP1)	chr2 120301847	cg23747904	− 0.55	− 4.17(0.27), − 3.67(1.37)	0.040	
		WD Repeat Containing. Antisense To TP73 (WRAP73)	chr1 3551807	cg04386615	− 0.54	3.66(0.33), 4.15(0.46)	0.040	
			chr1 1712021	cg23678594	− 0.34	− 4.31(0.19), − 3.98(0.43)	0.038	Promoter Associated
		Cyclin-Dependent Kinase 11B (CDK11B)	chr1 1655867	cg00669623	− 0.29	− 3.32(0.13), − 3.05(0.73)	0.037	Promoter Associated
		Solute Carrier Family 16 Member 11 (SLC16A11)	chr17 6947200	cg15639045	− 0.28	− 3.54(0.14), − 3.26(0.49)	0.011	
		Uncharacterized LOC284930	chr22 48182596	cg00331486	0.36	1.69(0.23), 1.35(0.59)	0.043	
		MyoD Family Inhibitor Domain Containing (MDFIC)	chr7 114562316	cg02239805	0.45	− 1.51(0.08), 1.96(1.42)	0.007	Promoter Associated
		G Protein-Coupled Receptor Kinase 4 (GRK4)	chr4 3033304	cg03178823	0.45	2.47(0.21), 1.99(0.93)	0.004	
		Mitogen-Activated Protein Kinase Kinase Kinase Kinase 5 (MAP4K5)	chr14 50999680	cg21062897	0.48	− 0.90(0.15), − 1.33(1.61)	0.036	Promoter Associated
			chr3 169476270	cg16056894	0.56	− 1.33(0.36), − 1.82(0.67)	0.038	
		Chromosome 10 open reading frame 11 (C10orf11)	chr10 77871958	cg24280832	0.94	1.48(0.39), 0.51(1.72)	< 0.000	

Location: chromosome and genomic position. Group difference in DNA methylation in specific CpGs are presented as log2 fold change (log2 (M-value group1/M-value group 2)) between the groups, meaning that a negative log2 fold changes means lower DNA methylation in group 1. Mean SD = mean standard deviation for group 1, group 2

chromosome (chr), standard deviation (SD)

In addition to specific DM CpGs, we also identified one differentially methylated region (DMR) when comparing HC with PND on SSRI treatment. The DMR included two CpGs located on chromosome 11 but in a unknown gene. Stratified analyses based on the sex of the child showed, among girls, one DMR in PND without SSRIs compared with PND treated with SSRI including two CpGs in the C10of11 gene. The exact same DMR was also identified in HC compared with PND treated with SSRIs. In boys, one DMR was identified in HC compared with PND treated with SSRIs, including two CpGs in the Family with sequence similarity 46 (FAM46B) gene.

We identified no DM CpGs within the genes of interest (*NR3C1*, *COL7A1*, *ZNF575*, *MEG3*, *SLC6A4*, *HSD11B2*, *FKBP5*, *BDNF*, *IGF2*, *PLAGL1*, *MEST,* and *PEG3*) based on previous findings in the literature in any of the group comparisons.

## Discussion

This study investigated the potential association between maternal PND with or without co-morbid anxiety, history of depression and SSRIs treatment with cord blood DNA methylation at birth. While no DM CpG site was identified in relation to PND alone, sub-group analyses showed differential DNA methylation associated with comorbidity with anxiety and SSRI treatment.

Prenatal depressive symptoms have been associated with less optimal development and associated alterations in child behavior, suggesting that it might influence fetal epigenetic programming [3]. In this study, no signs of aberrant fetal programming through epigenetic changes in cord blood were observed, since there were no DM found to be associated with PND alone. These negative findings are in agreement with most previous studies [16, 27, 32]. This could imply that PND, together with biological changes associated with this state, such as lower serum allopregnenolone [33], lower levels of peripheral anti-inflammatory markers [34] and lower levels of metabolites such as phosphate and arginine [35], do not affect DNA methylation in cord blood. However, it does not rule out other mechanisms through which PND could be involved in fetal programming effects, such as histone modifications and microRNAs, affecting gene expression. It could also be hypothesized that women with PND constitute a heterogeneous group, and that specific depressive phenotypes [8] need to be clearly outlined in order to find differences in epigenetic programming.

It has been proposed that earlier depression might have a persistent effect on biological systems even years after the depressive episode [9]. We therefore performed sub-group analyses, comparing PND alone with PND together with a history of depression. This sub-group analysis did not however show significant differences in DM based on history of depression. In contrast, the co-existence of PND with anxiety symptoms did show DM in one gene, *ABCF1*. Interestingly, *ABCF1* has been suggested to be one of the putative therapeutic targets for escitalopram, an SSRI treatment used for both depressive symptoms and anxiety [36]. Altered methylation of *ABCF1* has also been seen to be associated with autism in an in silico analysis multi-omics data analysis including frontal cortex samples [37]. Previous studies investigating the association between PND with anxiety symptoms and cord blood DNA methylation have not compared with PND alone compared with healthy controls [16, 38].

Of special interest are also the DM CpGs associated with SSRI treatment, especially located in or associated with the promoter region of specific genes. Variation in DNA methylation level in promoter regions is often associated with transcription factor binding where DNA methylation can affect the binding potential of transcriptional factors to promoter regions or transcription factor can protect the DNA from getting methylated. In both scenarios, a lower DNA methylation level is generally associated with more transcription factor binding and higher expression of the gene, higher DNA methylation in gene bodies is associated with increased gene expression. Many of the CpGs identified in the analysis in the SSRI-treated PND group are found in the promoter region of Cereblon (*CRBN)*, MyoD Family Inhibitor Domain Containing (*MDFIC),* Cyclin-Dependent Kinase 11B *(CDK11B), and* Mitogen-Activated Protein Kinase Kinase Kinase Kinase 5 *(MAP4K5) genes.* The *CRBN* gene has been shown to be involved in intellectual disabilities [39] and has immunomodulatory functions [40]. *MDFIC* has been shown to be involved in the cross-talk between the glucocorticoid receptor and other proteins important for the glucocorticoid responses [41]. The expression of MDIFC is also associated with the aggressiveness and clinical outcome in neuroblastoma [42], but the mechanisms are not well understood. *CDK11B* has been shown to be involved in neuroblastoma [43] while *MAP4K5* has been suggested to have a role in stress response [44]. Together, the DM of these genes, many related to stress and brain functions, in cord blood, might reflect altered methylation even in the fetal brain, with possible biological consequences in the child.

C10orf11 was one of the genes found to be DM in SSRI-treated PND group compared with both HC and PND without SSRIs. It was also identified as a DMR in girls in the SSRI-treated PND group. Interestingly, in rare occasions, disruption of C10ord11 has been associated with cognitive defects in children [45].

A previous study, by Cardenas and colleagues [16], also identified DM CpG sites in cord blood in relation to SSRI treatment during pregnancy; they did however not consider depressive symptoms with or without SSRI treatment. Amongst the CpG sites they identified, only one, *ZNF575,* was identified in their validation cohort. *ZNF575* was one of the genes of interest separately analyzed even in present study, but no DM was observed in this gene in our cohort. Further, Cardenas and colleagues did not perform any stratified analysis by the sex of the child, while our analysis showed substantial differences between the sexes. Female offspring had considerably more DM sites between groups in comparison with the whole cohort and male offspring. It is however important to note that both the study by Cardenas and colleagues [16] and our study are very limited by the small sample size, and all results must be further validated in larger study cohorts.

Maternal SSRI use during pregnancy has been shown to affect development in the child. However, the effects of SSRI treatment during pregnancy are difficult to investigate without taking into account the underlying effects of depressive symptoms as reviewed by Olivier et al. 2013 [46]. We have previously shown that there are differences in gene expression in the placenta of women with PND being treated with SSRI compared with untreated women with PND [12]. Also, women with PND treated with SSRI have been shown to have higher corticotropin-releasing hormone levels associated with increased risk of pre-term birth [11] compared with untreated women with PND. Our findings add on to the knowledge that there are different biological effects between women experiencing PND with and without SSRI treatment, but it is impossible to distinguish if these are due to the treatment itself, a more severe PND demanding SSRI treatment, or a more prolonged depressive episode. Larger studies with a large number of women with severe depression without medication should be further encouraged. Unfortunately, this is not easy as it is not ethical not to treat depression, and women choosing themselves not to take medication might differ from the rest of the participants.

Strengths of the present study are the population-based prospective assessment of different sub-types of PND with EWAS analysis in cord blood. Moreover, weaknesses of previous studies were addressed in the current study through adjustment for mode of delivery [47] and cell composition known to affect DNA methylation [28, 29], as well as the exclusion of participants with pregnancy complications, also known to affect DNA methylation [48, 49]. Further, we have implemented strict control for multiple testing and stratified our analyses by sex known to affect DNA methylation [30] but also the effect by PND on the child [3]. The results should also be considered in relation to the following limitations: the relatively small sample size, albeit not uncommon in EWAS analyses. Future studies may consider joining the available cohorts investigating the effect of SSRI treatment on cord blood DNA methylation in a meta-analysis in order to gather more statistical strength and perform permutation analysis in a greater cohort. The BASIC study’s relatively low participation rate [50] makes the results not readily generalizable to the background population also depression was assessed through self-reports on a screening instrument and not a clinical diagnosis. In addition, the magnitude of differences in methylation levels was small between groups. The use of surrogate tissue is also a limitation. The cell/tissue-specificity of epigenetic patterns is a major challenge in psychological epigenetics. However, cell-specific analysis would limit research to certain brain areas of suicide victims or cadavers and limit the possibility of investigating early biomarkers affected by maternal depressive symptoms. DNA methylation in blood has been found to be in good agreement with DNA methylation in brain, especially when comparing individual CpGs [22]. Furthermore, we have highlighted in the discussion genes with relevance in stress and brain function with possible biological consequences in the child. In addition, we did not perform any adjustment for cross-reactive probes before group comparisons, which can cause false signals, possibly resulting in invalid conclusions. One of our DM probes were included on the list of genes previously identified cross-reactive probes [51], and this has now been removed from the presented list of DM genes, which should account for this limitation. Finally, this study did not investigate longitudinal follow-up of child behavior in relation to cord blood DNA methylation.

In conclusion, this study investigated potential associations between maternal PND with child epigenetic markers at birth, taking into account history of depression, comorbid anxiety or SSRI treatment, and also sex of the child. While no DM CpG site was identified in relation to PND alone, differences in DNA methylation were observed in the cord blood of children whose mothers had PND together with anxiety and also those who were treated with SSRIs. Several genes, for instance *CRBN* and *MDFIC*, previously shown to be associated with brain development and function where identified in those who were exposed to SSRIs. This study can however not distinguish between the effects of SSRI treatment alone or a possibly more severe PND demanding SSRI treatment. Furthermore, the findings must be validated in larger study cohorts including more SSRI exposed samples.

## Methods

### Study population

This study includes mothers and offspring participating in the BASIC Study (“Biology, Affect, Stress, Imaging and Cognition in pregnancy and puerperium”), a longitudinal study that recruited patients between 2009 and 2019 with the aim to enhance the understanding of perinatal depression [50].

All pregnant women in Uppsala County, over 18 years of age, who speaks Swedish and were scheduled for a routine ultrasound at Uppsala University Hospital, were invited to participate in the BASIC study. Women diagnosed with a pathological pregnancy (malformations leading to termination of pregnancy or miscarriage), with blood-borne disease, or with protected personal data were excluded. The participation rate has been estimated to be 22% of all women giving birth in Uppsala County [50]. Participants contributed via web-based questionnaires during gestational weeks 17 and 32 and postpartum at six weeks, six months, and twelve months. Umbilical cord blood samples were collected from participants during childbirth.

At gestational week 17, participants reported their age, education, smoking habits, employment, and country of birth (a proxy for ethnicity) by use of web-based questionnaire. Participants were also asked questions about their history of depression. Medical information regarding the pregnancy and birth was extracted from medical records.

The Edinburgh Postnatal Depression Scale (EPDS), validated in Swedish [52, 53], was included in the web-based questionnaires at pregnancy week 17 and 32. Anxiety symptoms were measured with the State-Trait Anxiety Inventory for Adults (STAI-AD, state scale) or the Beck Anxiety Inventory (BAI), depending on the time point of inclusion to the BASIC study [50].

For this sub-study, mothers were included if they had been followed throughout pregnancy and the postpartum period until six months postpartum, and a cord blood sample was collected at birth (*n* = 2,595), as described in Fig. 1. Exclusion criteria were women who were smoking as smoking affects epigenetic modifications. In addition, as samples were collected for additional analyses of child behavior at later in life, women presenting depressive symptoms in the postpartum period were excluded as postpartum depression could affect offspring psychoemotional development.

Only one pregnancy was included if women had participated multiple times in BASIC, and if the mother experienced depressive symptoms in one pregnancy but not the other, the one with depressive symptoms was chosen, otherwise the first pregnancy was included. Mothers defined as controls had EPDS ≤ 11 at both pregnancy week 17 and 32, had to have answered all questionnaires throughout the study and were excluded if they had anxiety defined by use of STAI, BAI or the EPDS anxiety subscale, any history of depression, or had missing information from medical records. Women with depressive symptoms during pregnancy were identified by the use of EPDS > 12 at pregnancy week 17, pregnancy week 32 or both. Final analyses also excluded women with pregnancy complications such as pregnancy hypertension, diabetes, itching, placenta previa, anemia, hepatosis, cystitis, preeclampsia, or HELLP syndrome. This resulted in 373 pregnancies included in the final analysis (Fig. 1).

Mothers were divided into two main study groups, mothers with PND (*n* = 178) or healthy controls (HC) (*n* = 195). Sub-groups analyses were also performed between women with PND with (*n* = 132) or without history of depression (*n* = 46), in comparison with HC, as well as PND comorbid with anxiety (*n* = 101) or not (*n* = 77), in comparison with HC. Finally, also based on PND with (*n* = 13) or without SSRI treatment (*n* = 168) during pregnancy, in comparison with HC.

### Cord blood collection and DNA extraction

In all steps, from DNA extraction to DNA methylation analysis, PND and HC samples were randomized over batches and arrays. Whole blood from the cord vein was collected at childbirth and stored at − 70 °C until DNA extraction by use of QIAamp DNA blood/mini kit (Qiagen) according to manufacturer’s instructions. DNA concentrations and purity were measured by use of NanoDrop Spectrophotometer (Thermo Scientific).

### DNA methylation data

Bisulfite conversion of DNA was performed by use of the Zymo Research DNA Methylation-Lightning Kit (Zymo Research). Genome-wide methylation at 850 000 CpG sites was subsequently analyzed with the Illumina MethylationEPIC kit (Illumina). All reactions were performed according to the manufacturers' instructions at Life and Brain Life & Brain Research Centre University Hospital of Bonn, Germany. Data processing was carried out by National Bioinformatics Infrastructure Sweden (NBIS) in R 3.5.2 using R package minfi 1.28.4 [54]. The workflow followed was as per recommended by the tool guide. Detection *p*-value (DPV) with a cut-off at 0.01 was used to remove CpGs with poor signal (4.5% of the CpGs had DPV > 0.01 in 10% or more of the samples). No samples, only individual CpGs were removed based on DPV. An additional 3.2% of the CpGs were discarded due to presence of known SNPs in the CpG site or single-base extension. SNP annotation data were obtained through minfi:getSnpInfo, which retrieves SNP data from IlluminaHumanMethylationEPICanno.ilm10b2.hg19 version 0.6.0. All sites with SNPs were removed as recommended by the supplier of the Illumina MethylationEPIC kit (Illumina).

Functions in the R package minfi were used for quantile normalization suitable for datasets where global differences are not expected between samples [55]. Sex information was provided to the function rgf_quantile  ← preprocessQuantile(rgf,sex = sx), beta_quantile ← getBeta(rgf_quantile), m_quantile ← get_mv(beta_quantile). This implements a stratified quantile normalization which is applied to the methylated and unmethylated signal intensities separately and takes into account the different probe types [56]. This to correct for non-biological variation between CpGs and the data was thereafter transformed to beta, representing the proportion of cells methylated at the each CpG site (from 0 to 1 methylation) and M values for statistical analysis. B-values were chosen for visualization as recommended in the literature [57]. CpGs from X and Y chromosomes were discarded and only autosomal CpGs were used for downstream analyses, according to the tool guide instructions. Cell composition in each sample was estimated using R package FlowSorted.CordBlood.450 k [29] as the reference data.

### Statistical analyses

PCA was used for exploratory analyses to investigate general structure and patterns in the data. Beta values were used for PCA. The 100,000 CpGs with the highest variance were selected for the PCA analyses. Up to 17% of the total variation in the dataset was explained by the first three principal components (PCs). Benjamini–Hochberg corrected Kruskal–Wallis *p*-values were used a measure to assess association of top 6 PCs with categorical variables of interest. Benjamini–Hochberg corrected Spearman correlation *p*-values were used a measure to assess association of top 6 PCs with continuous variables of interest.

Differential methylation (DM) CpGs between the study groups and sub-groups were identified using the R package Limma. The data were also stratified by sex of the child and the analysis was also run excluding participants using SSRI. For each dataset, DM tests were run to assess how many CpGs differed significantly in methylation between various groups. M values were used for DM analysis. For each test, a linear model was built including the variable of interest along with 12 covariates, which were controlled for in the model. The following covariates were used in all the models: sample plate batch, sentrix position on the chip, granulocyte cell estimate, red blood cell estimate, CD8T cell estimate. Also clinical characteristics such as maternal age at delivery, BMI before pregnancy, maternal place of birth (Scandinavia or other), educational level (University degree yes or no), mode of delivery (vaginal, vacuum extraction, emergency cesarean section, and elective cesarean section), gestational age, and parity, based on previous literature [16, 47, 58, 59]. Group differences in DNA methylation in specific CpGs are presented as mean and standard deviation and also log2 fold change (log2 (M-value group1/M-value group 2)) between the groups, meaning that a negative log2 fold changes means lower DNA methylation in group 1. Pathway enrichment analysis on DM genes was performed by use of the FUMA tool by using the mode GENE2FUNC [60].

Differential methylation over regions was run using R package DMRcate 2.4.1 in R 4.0.2 [61]. The parameters (lambda = 500, C = 5) were set as recommended in Mallik et al., 2019 [62]. An FDR cut-off of 0.05 was used to determine significance.

In addition to the overall comparison of all DM between groups, CpGs in genes of interest identified in the literature [16, 18, 25, 26] and were included in a separate analysis, in which the adjustment of *p*-value for multiple comparisons was based on the number of CpGs in the genes of interest, instead of all included in the EPIC array. These genes of interest were: Nuclear Receptor Subfamily 3 Group C Member 1 (*NR3C1*), Collagen Type VII Alpha 1 Chain (*COL7A1*), Zinc Finger Protein 575 *(ZNF575*), Maternally Expressed Gene 3 (*MEG3*), Solute Carrier Family 6 Member 4 (*SLC6A4*), Hydroxysteroid 11-Beta Dehydrogenase 2 (*HSD11B2*), FKBP Prolyl Isomerase 5 (*FKBP5*), Brain Derived Neurotrophic Factor (*BDNF*), Insulin Like Growth Factor 2 (*IGF2*), PLAG1 Like Zinc Finger 1 (*PLAGL1*), Mesoderm Specific Transcript (*MEST*) and Paternally Expressed 3 (*PEG3*) [16, 18, 25, 26].

## Supplementary Information

**Additional file 1. Figure S1:** Quantile-quantile plot (QQ-plot) displaying the observed and expected Benjamini-Hochberg adjusted –log10 p-values for all group comparisons. a) Healthy control versus Prenatal depressive symptoms. b) History and prenatal depressive symptoms versus Prenatal depressive symptoms only. c) History and prenatal depressive symptoms versus Healthy control. d) Prenatal depressive symptoms only versus Healthy control. e) Anxiety and prenatal depressive symptoms versus Prenatal depressive symptoms only. f) Anxiety and prenatal depressive symptoms versus Healthy control. g) Prenatal depressive symptoms only versus Healthy control. h) Healthy control versus Prenatal depressive symptoms without selective serotonin reuptake inhibitors (SSRI). i) Healthy control versus Prenatal depressive symptoms with SSRI. j) Prenatal depressive symptoms without SSRI versus Prenatal depressive symptoms with SSRI.

**Additional file 2. Figure S2:** Manhattan plots displaying differentially DNA methylated genes in cord blood from infants born by mothers who a) suffer from anxiety and prenatal depressive symptoms compared with healthy controls, b) healthy controls compared with prenatal depressive symptoms treated with selective serotonin reuptake inhibitors (SSRIs), and c) women with untreated prenatal depressive symptoms compared with prenatal depressive symptoms treated with SSRIs. Y-axes display the Benjamini-Hochberg adjusted –log10 p-values for specific group comparisons and x-axes shows the chromosomal location.

## Data Availability

The data that support the findings of this study are available from the authors upon reasonable request and with permission of BASIC study cohort.
