# Supplementary material for: DNA methylation in cord blood in association with prenatal depressive symptoms
Source: Clin Epigenetics. 2021 Apr 12;13:78. doi: 10.1186/s13148-021-01054-0 (PMC8042709; doi:10.1186/s13148-021-01054-0)
Supplement: Supplementary file 1 — Additional file 1. Figure S1: Quantile-quantile plot (QQ-plot) displaying the observed and expected Benjamini-Hochberg adjusted –log10 p-values for all group comparisons. a) Healthy control versus Prenatal depressive symptoms. b) History and prenatal depressive symptoms versus Prenatal depressive symptoms only. c) History and prenatal depressive symptoms versus Healthy control. d) Prenatal depressive symptoms only versus Healthy control. e) Anxiety and prenatal depressive symptoms versus Prenatal depressive symptoms only. f) Anxiety and prenatal depressive symptoms versus Healthy control. g) Prenatal depressive symptoms only versus Healthy control. h) Healthy control versus Prenatal depressive symptoms without selective serotonin reuptake inhibitors (SSRI). i) Healthy control versus Prenatal depressive symptoms with SSRI. j) Prenatal depressive symptoms without SSRI versus Prenatal depressive symptoms with SSRI. [file 13148_2021_1054_MOESM1_ESM.docx]

1.
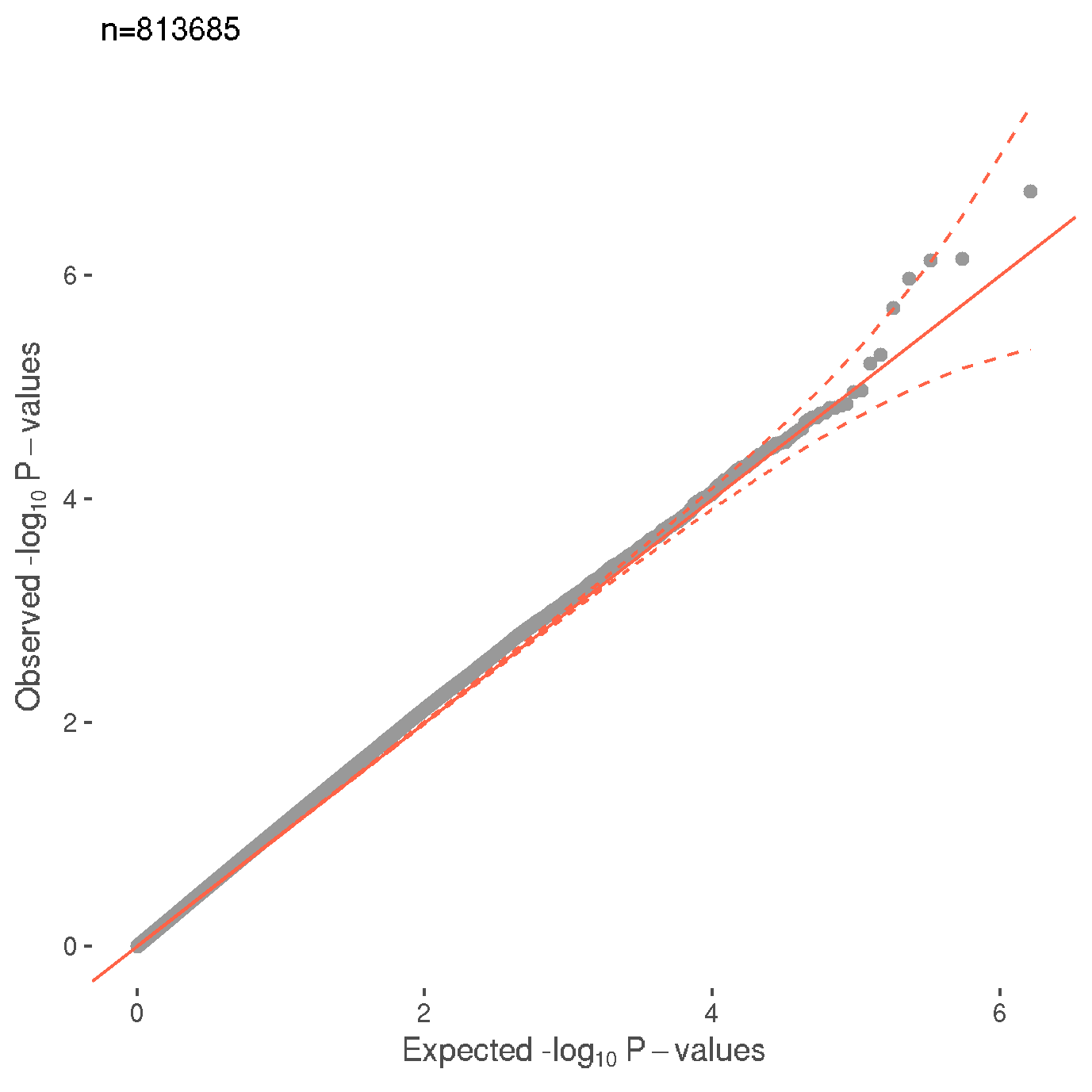
b)
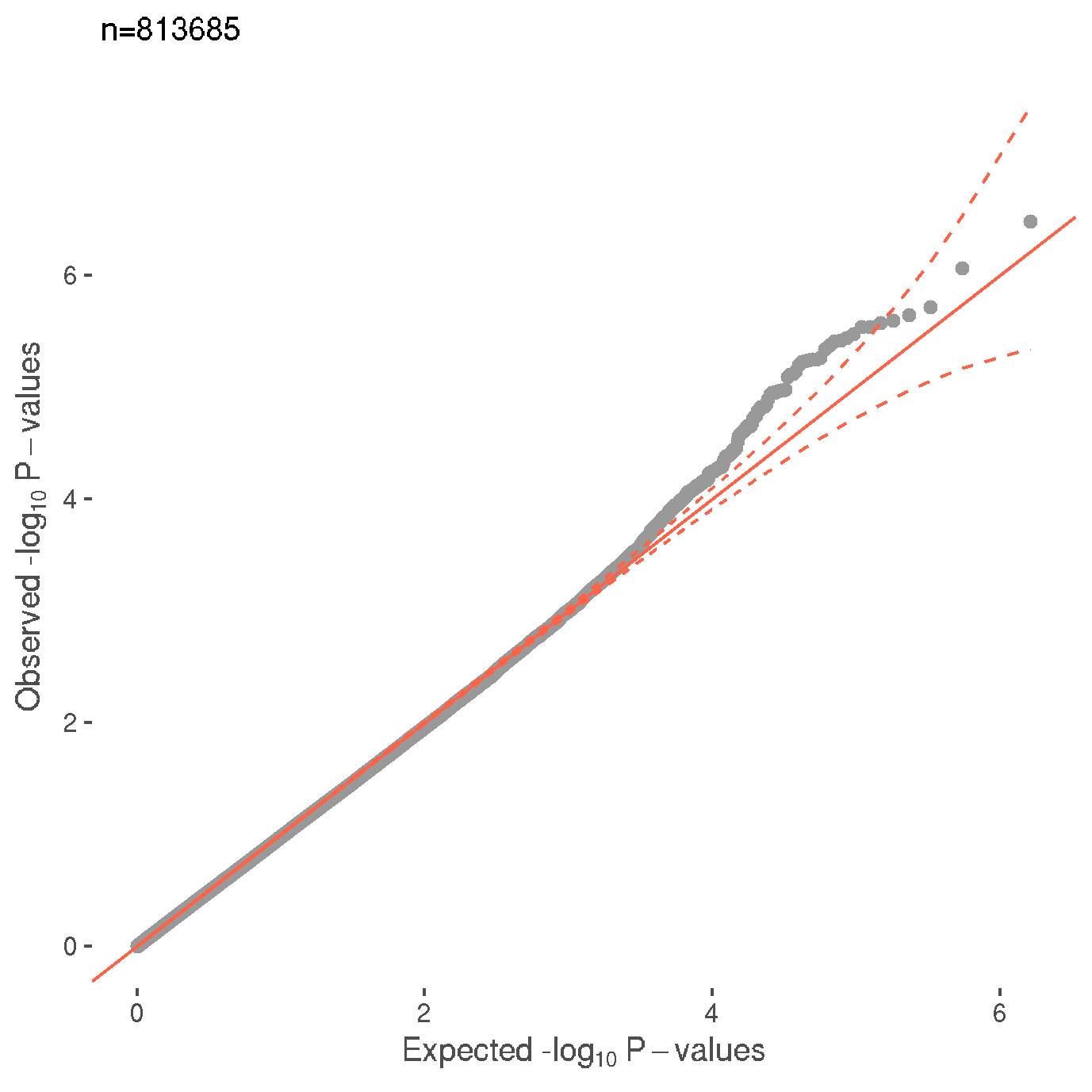


c)
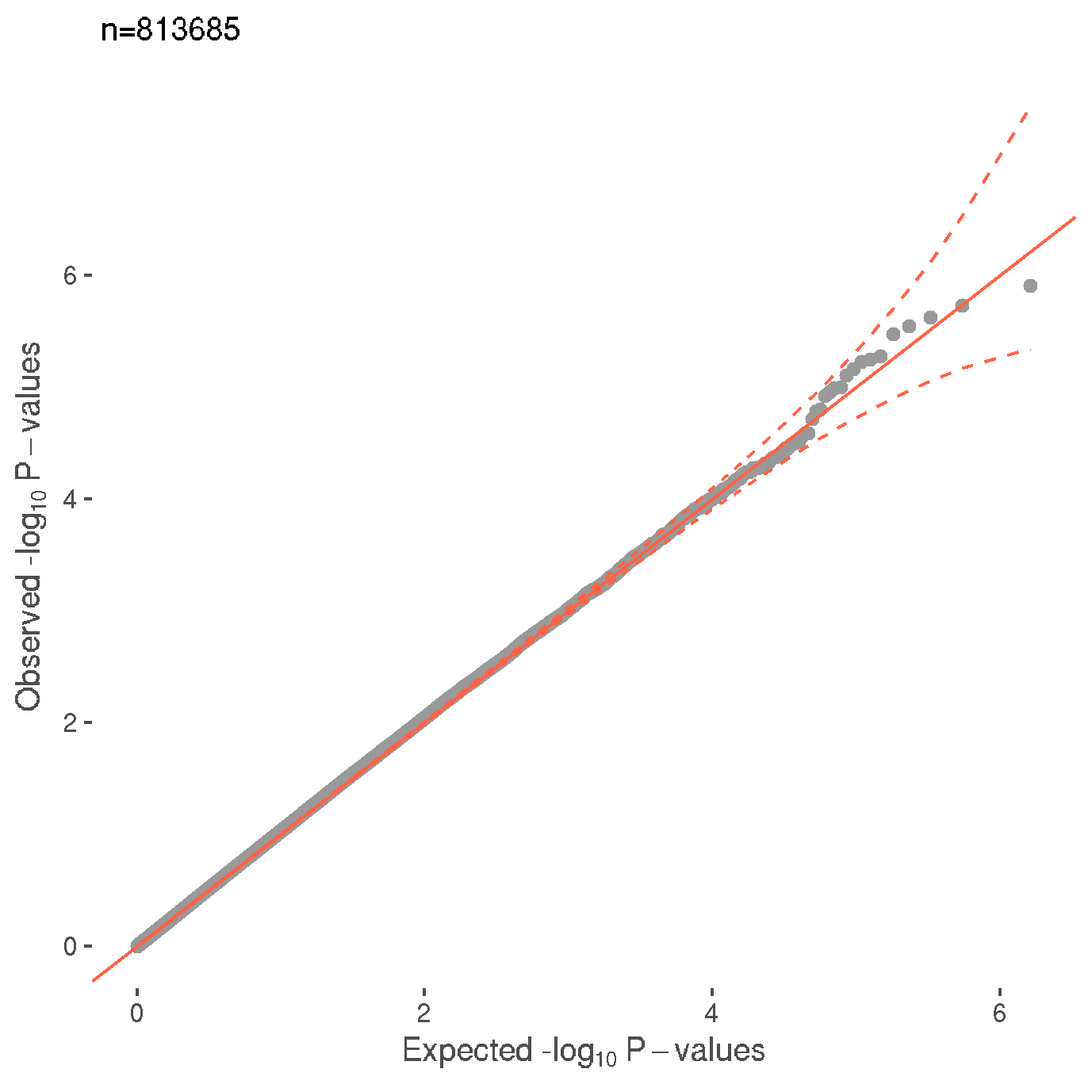
d)
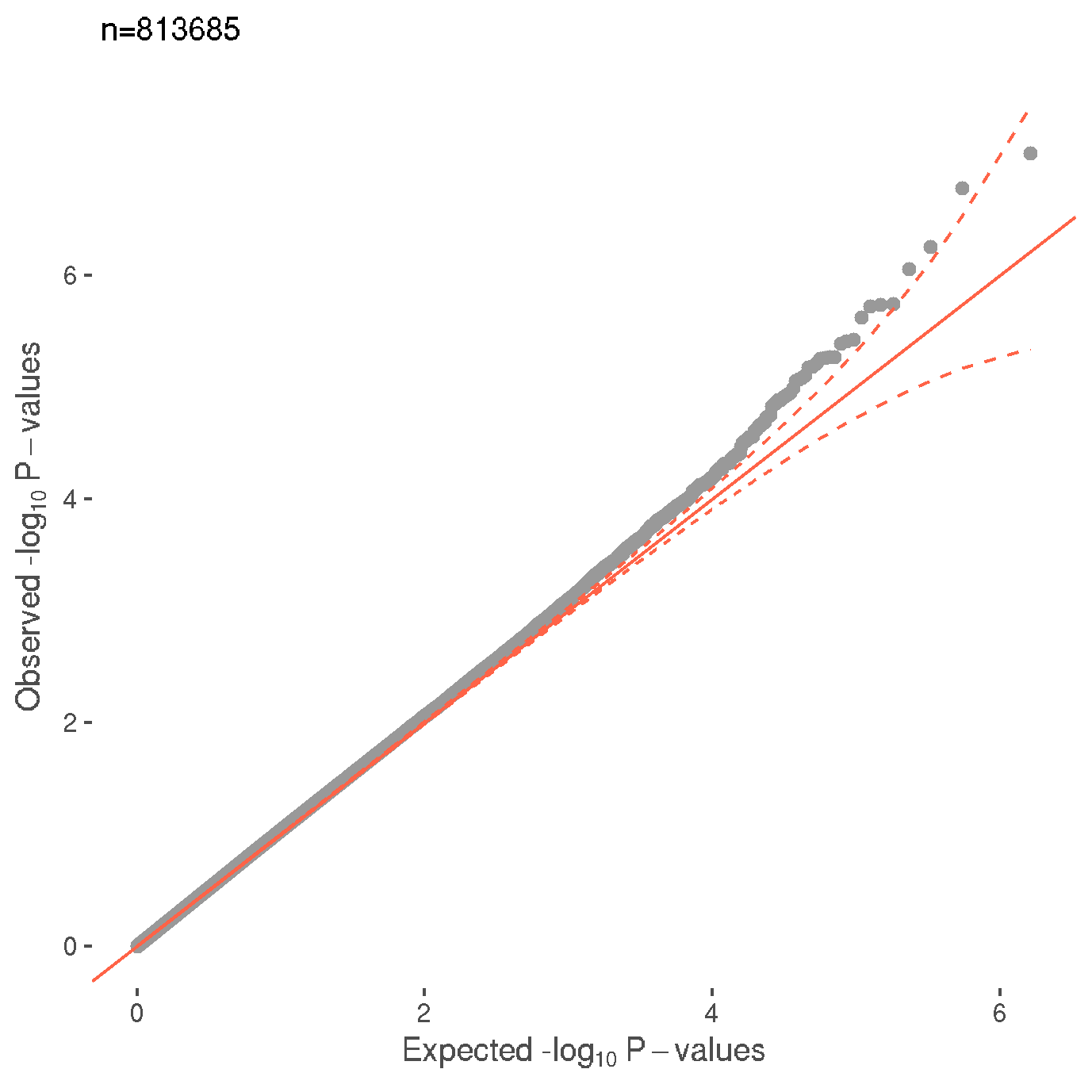


e)
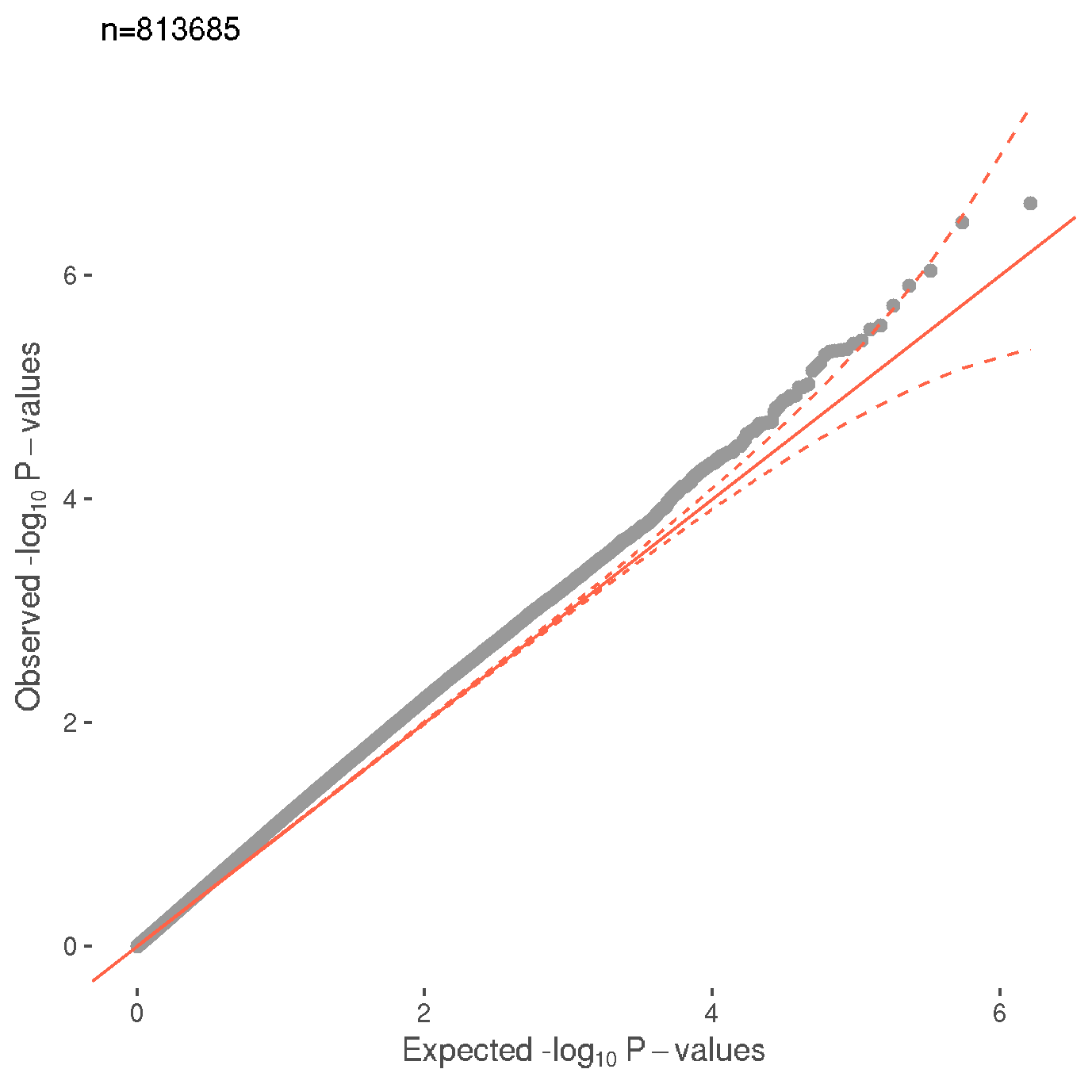
f)
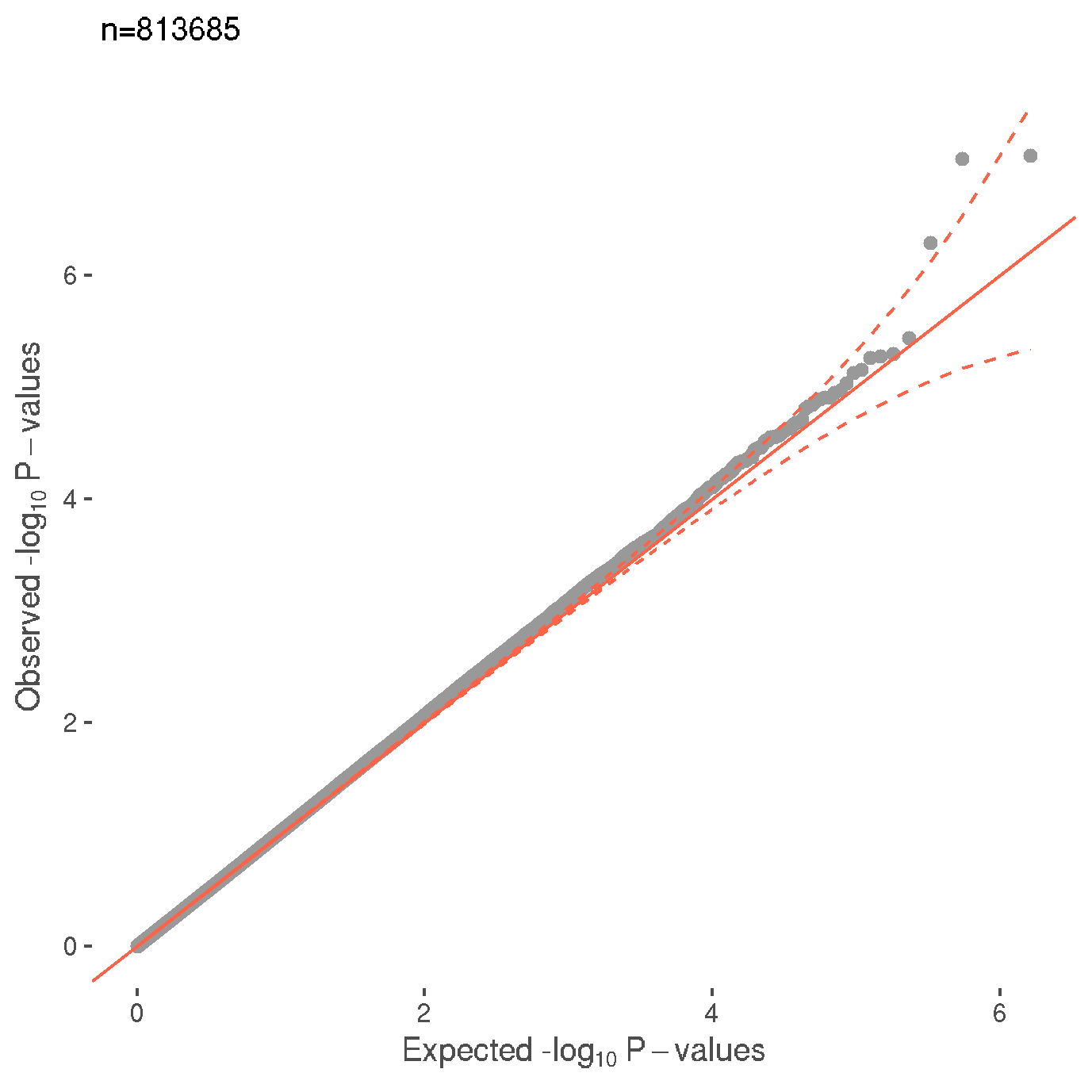


g)
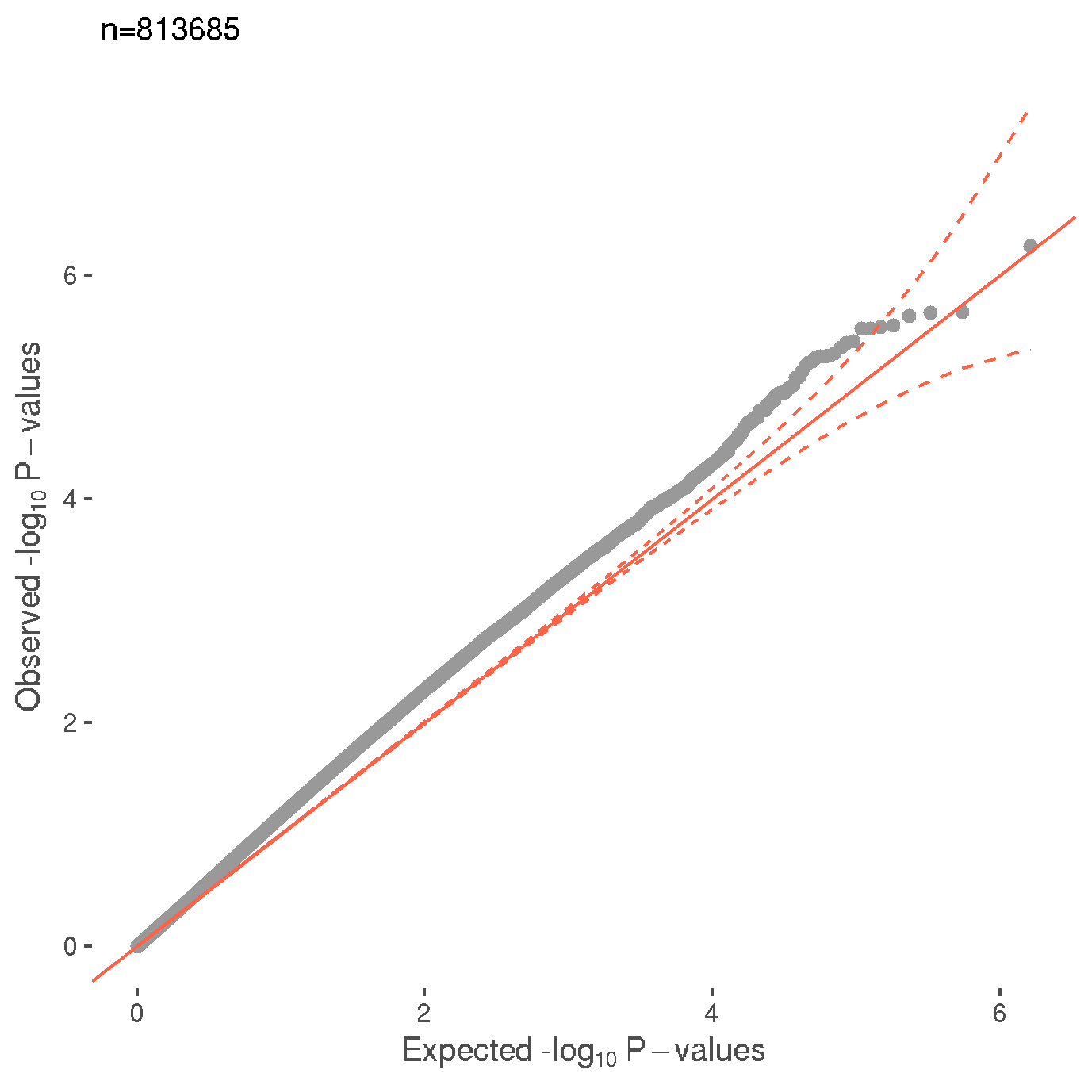
h)
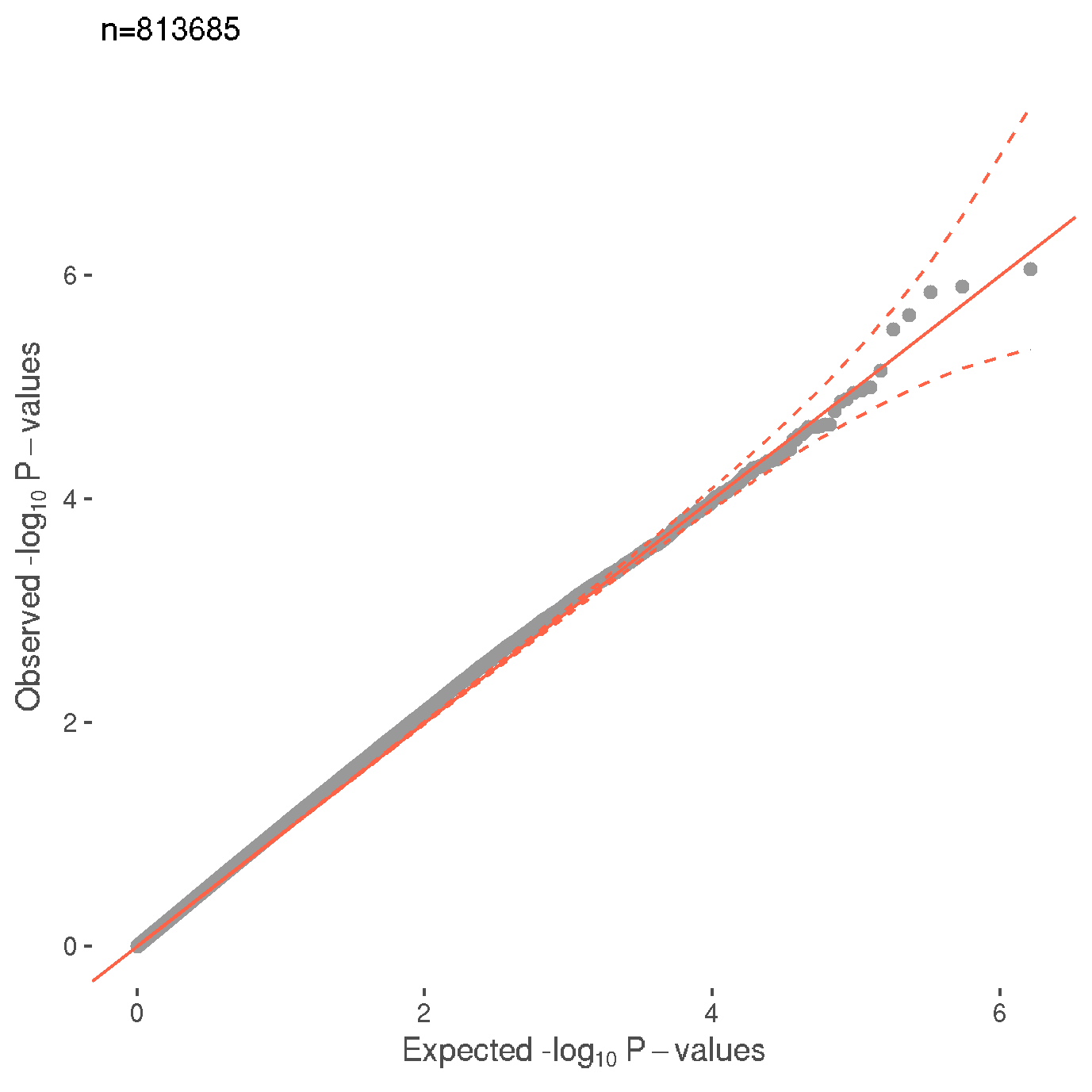


i)
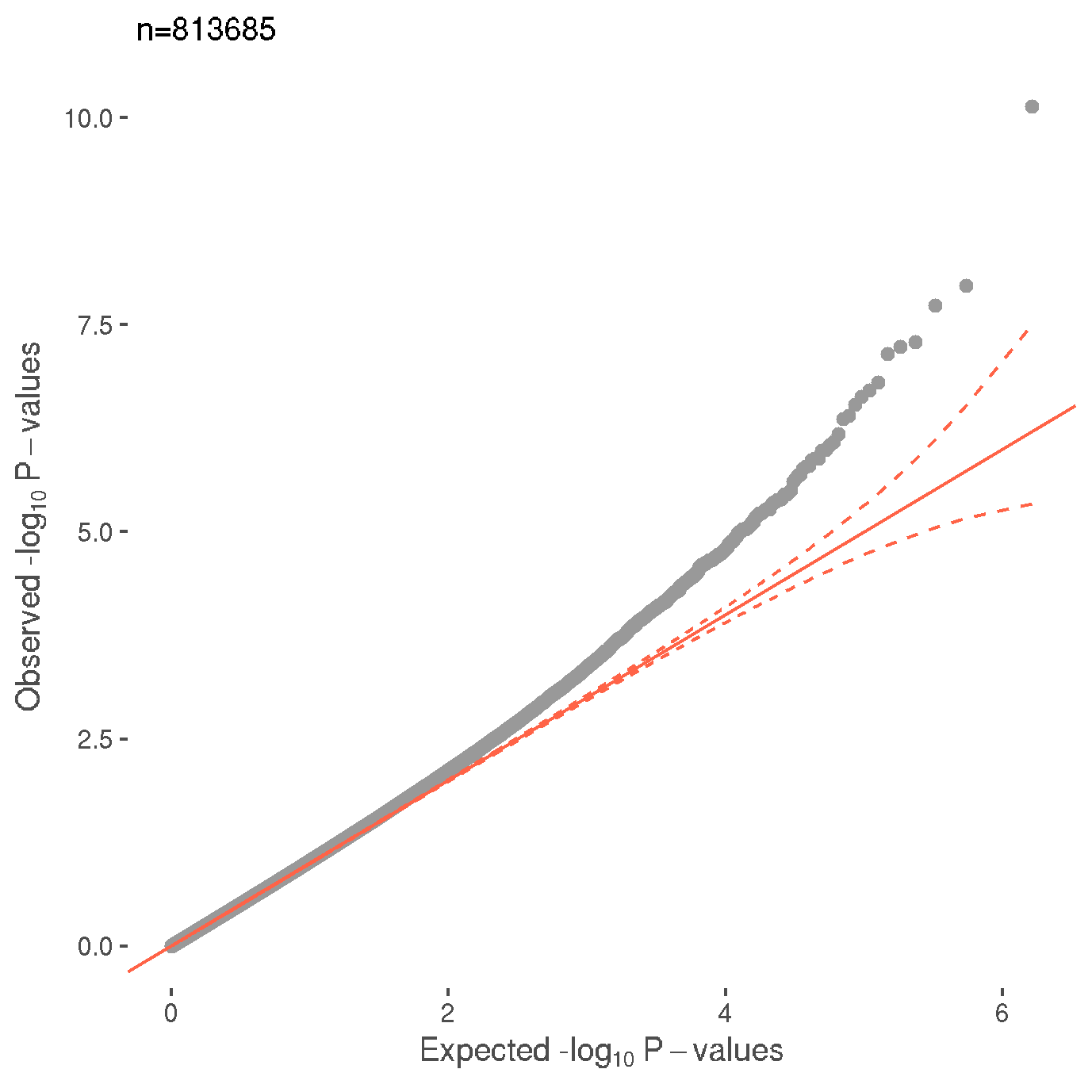
j)
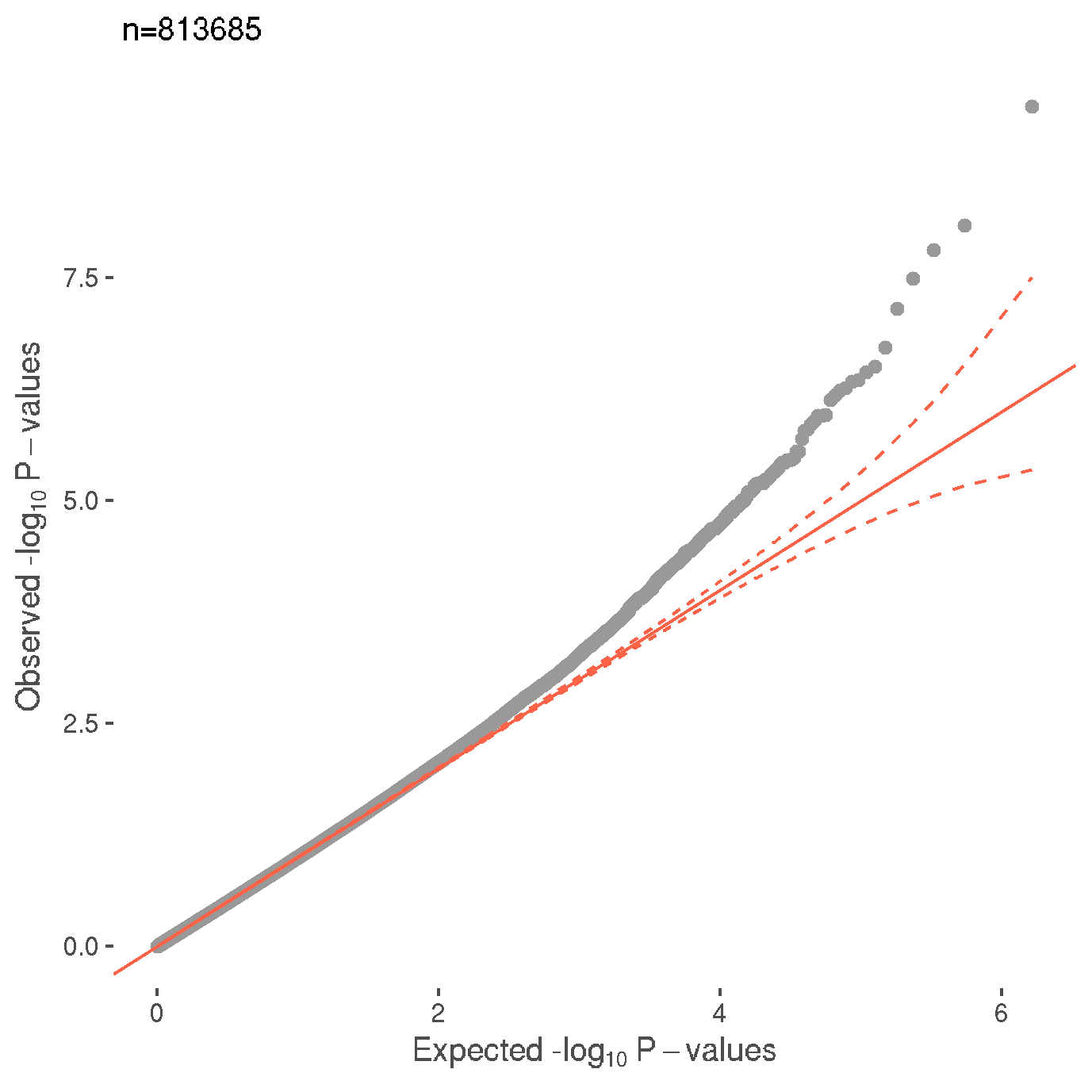


Supplementary figure 1.

| Quantile-quantile plot (QQ-plot) displaying the observed and expected Benjamini-Hochberg adjusted –log_10_ p-values for all group comparisons.  a) Healthy control versus Prenatal depressive symptoms |
| --- |
| b) History and prenatal depressive symptoms versus Prenatal depressive symptoms only |
| c) History and prenatal depressive symptoms versus Healthy control |
| d) Prenatal depressive symptoms only versus Healthy control |
| e) Anxiety and prenatal depressive symptoms versus Prenatal depressive symptoms only |
| f) Anxiety and prenatal depressive symptoms versus Healthy control |
| g) Prenatal depressive symptoms only versus Healthy control |
| h) Healthy control versus Prenatal depressive symptoms without SSRI |
| i) Healthy control versus Prenatal depressive symptoms with SSRI |
| j) Prenatal depressive symptoms without SSRI versus Prenatal depressive symptoms with SSRI |
