# Supplementary material for: DNA methylation in cord blood in association with prenatal depressive symptoms
Source: Clin Epigenetics. 2021 Apr 12;13:78. doi: 10.1186/s13148-021-01054-0 (PMC8042709; doi:10.1186/s13148-021-01054-0)
Supplement: Supplementary file 2 — Additional file 2. Figure S2: Manhattan plots displaying differentially DNA methylated genes in cord blood from infants born by mothers who a) suffer from anxiety and prenatal depressive symptoms compared with healthy controls, b) healthy controls compared with prenatal depressive symptoms treated with selective serotonin reuptake inhibitors (SSRIs), and c) women with untreated prenatal depressive symptoms compared with prenatal depressive symptoms treated with SSRIs. Y-axes display the Benjamini-Hochberg adjusted –log10 p-values for specific group comparisons and x-axes shows the chromosomal location. [file 13148_2021_1054_MOESM2_ESM.docx]

**a)**
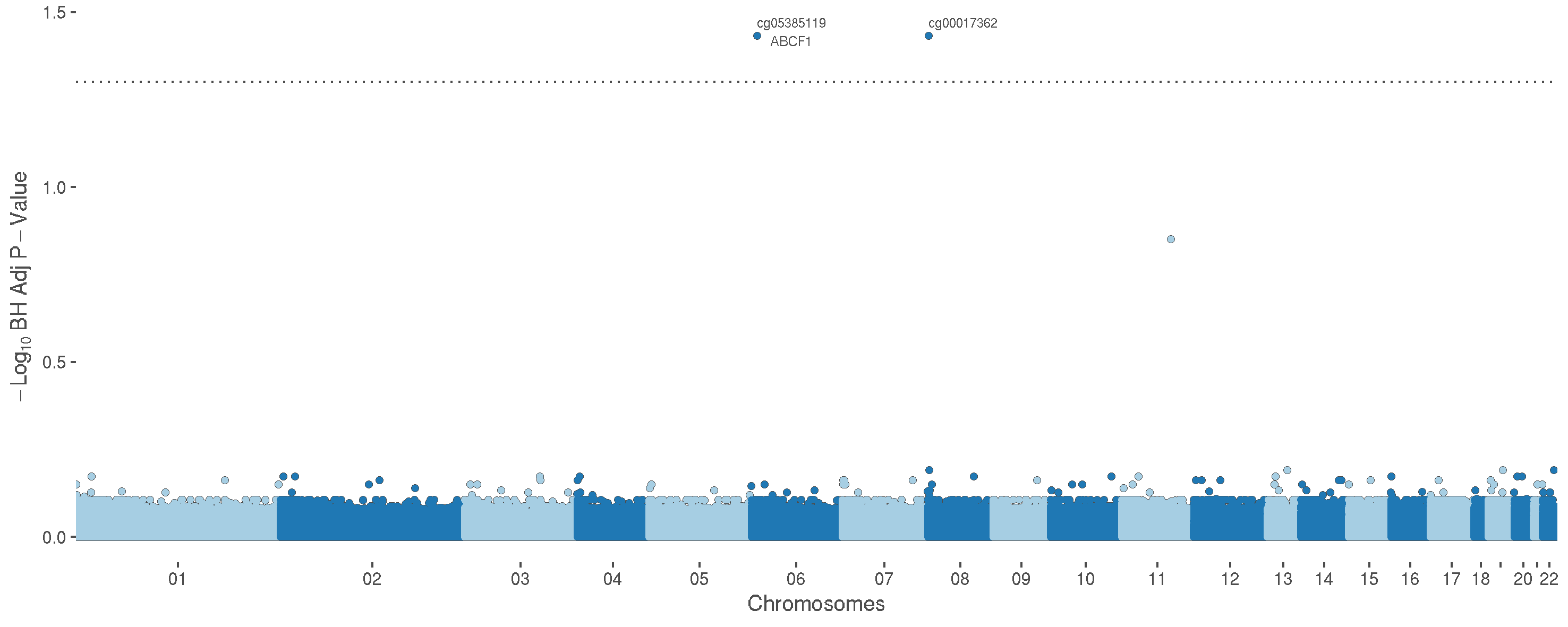


**b)**


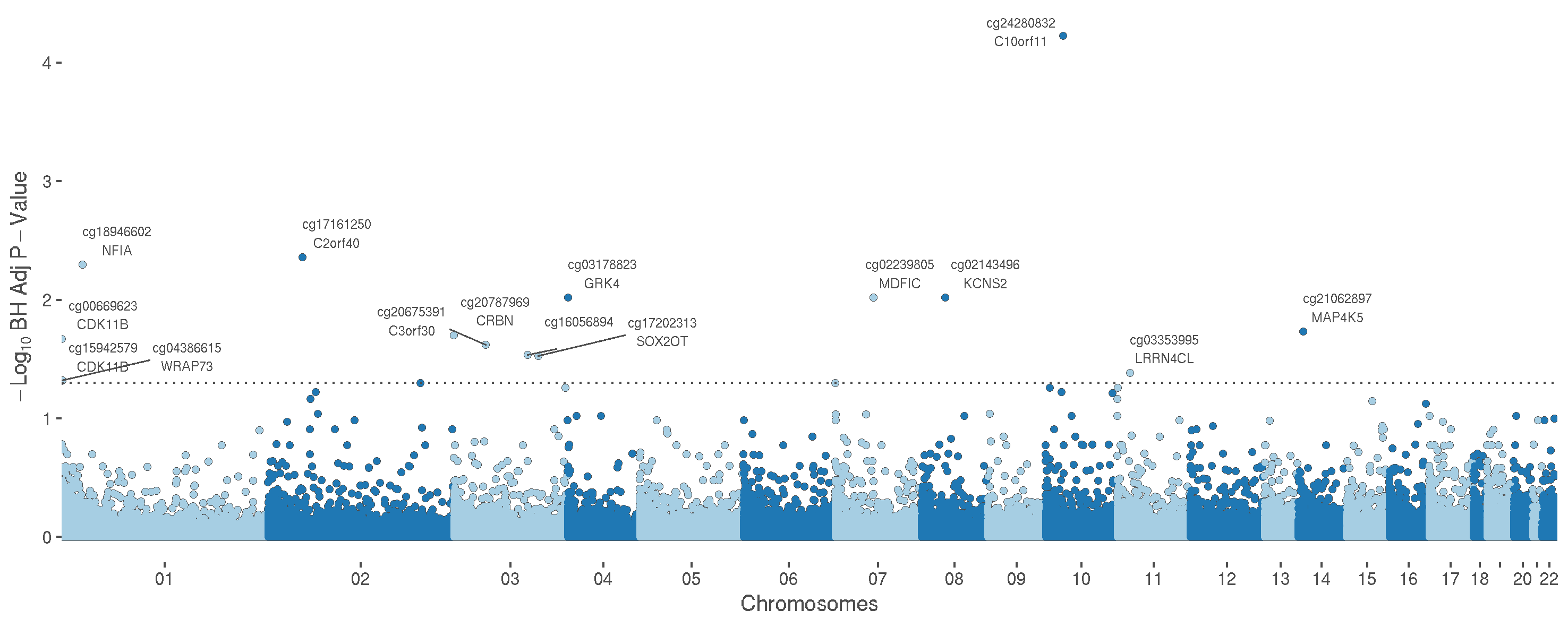


**c)**


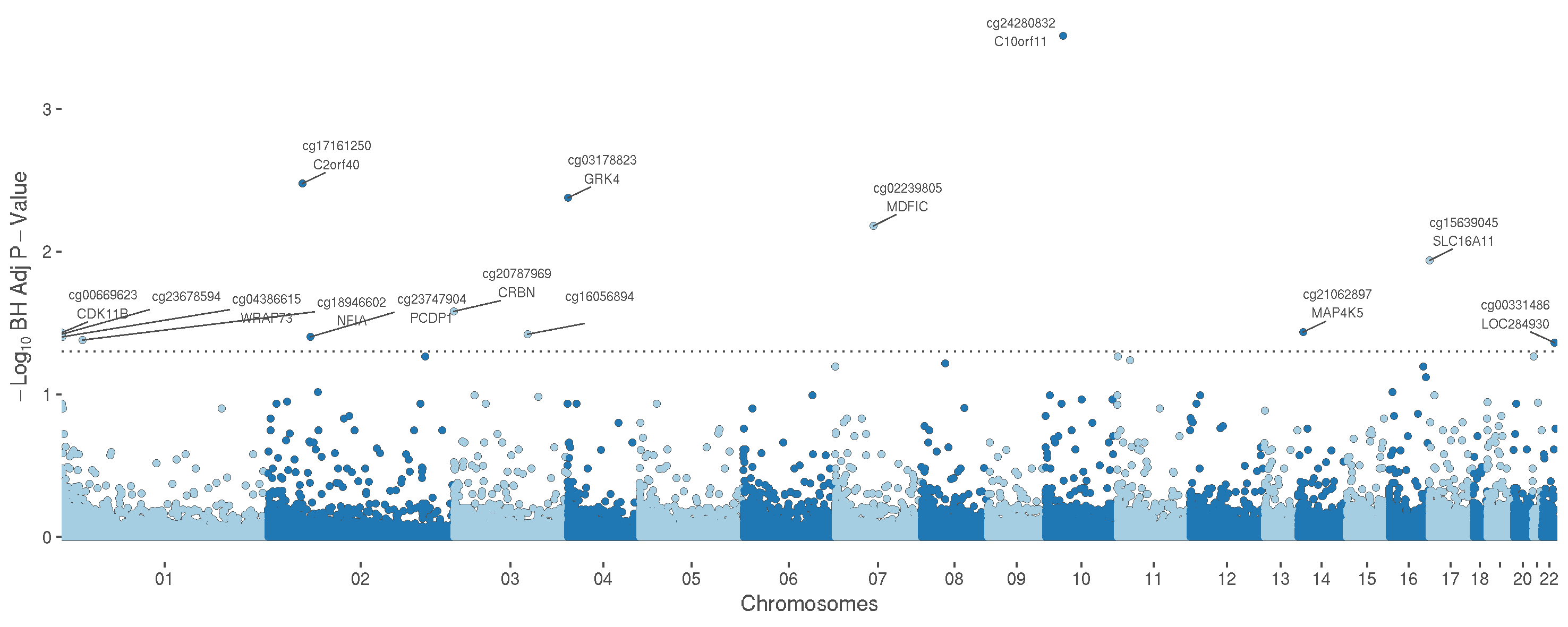


Supplementary figure 2.

Manhattan plots displaying differentially DNA methylated genes in cord blood from infants born by mothers who a) suffer from anxiety and prenatal depressive symptoms compared with healthy controls, b) healthy controls compared with prenatal depressive symptoms treated with selective serotonin reuptake inhibitors (SSRIs), and c) women with untreated prenatal depressive symptoms compared with prenatal depressive symptoms treated with SSRIs. Y-axes display the Benjamini-Hochberg adjusted –log_10_ p-values for specific group comparisons and x-axes shows the chromosomal location.
